# Optic Nerve Head Curvature Flattening Is Associated with Central Visual Field Scotoma

**DOI:** 10.3390/jcm13020596

**Published:** 2024-01-19

**Authors:** Keunheung Park, Jinmi Kim, Jiwoong Lee

**Affiliations:** 1 Department of Ophthalmology, Busan Medical Center, Busan 47527, Republic of Korea; 2 Department of Biostatistics, Clinical Trial Center, Biomedical Research Institute, Pusan National University Hospital, Busan 49241, Republic of Korea; 3 Department of Ophthalmology, Pusan National University College of Medicine, Busan 50612, Republic of Korea; 4 Biomedical Research Institute, Pusan National University Hospital, Busan 49241, Republic of Korea

**Keywords:** myopia, glaucoma, optic nerve head

## Abstract

This study aimed to develop a new index, the average curvature ratio (ACR), to represent the optic nerve head (ONH) tilting and investigate its clinical relevance. Myopic eyes were included and divided into two subgroups: flat ONH (ACR < 1.0) and convex ONH (ACR ≥ 1.0). The occurrences of central and peripheral visual field (VF) defects were compared between the two groups. A total of 375 myopic eyes were recruited, and 231 and 144 eyes were included in the flat and convex ONH groups, respectively. Central scotoma occurred more frequently in the flat ONH group. According to the Patella–Anderson criteria, the number of eyes with central scotoma was 103 (44.6%) in the flat and 44 (30.6%) in the convex ONH groups (*p* = 0.009). According to Kook’s criteria, the number of eyes with central scotoma was 122 (52.8%) in the flat and 50 (34.7%) in the convex ONH groups (*p* < 0.001). Peripheral scotoma was not significantly different between the groups. In the correlation analysis, the ACR was positively correlated with spherical equivalence, but not with axial length or central corneal thickness. The ACR reflects the degree of the ONH tilt and is a good index for estimating central VF damage in myopic eyes.

## 1. Introduction

Myopia is a highly frequent eye disease, especially in Asia, with a prevalence of 80–90% [1]. The prevalence of myopia among young adults is approximately 10–20% [2], and it usually does not cause blindness, but is a well-known risk factor for the development of glaucoma [3,4]. It causes central visual field (VF) defects [5] and greatly affects the quality of life. Myopia is characterized by structural changes, including the changes in the optic nerve head (ONH) caused by the elongation of the eyeball [6]. Structural changes are thought to contribute a lot of stress on the eyes and increase the risk of glaucoma [7,8].

The ONH tilting is one of the most frequently observed morphological changes in myopia [9]. Chang et al. [9] reported that the ONH tilting is the second most common structural change following peripapillary atrophy (PPA), and is present in 57.4% of highly myopic eyes in adults. With the rapid development of ocular imaging equipment, in-depth research has been conducted on structural changes in the myopic ONH [5,10,11]. Studies have revealed that the ONH tilting is related to retinal nerve fiber layer (RNFL) thinning, lamina cribrosa (LC) defects, lower visual acuity, and worse central VF defects [5,10,11].

To measure the amount of ONH tilting, several studies have used optical coherence tomography (OCT) [12,13,14]. This method has the advantage of clearly identifying the peripapillary structures, such as the Bruch’s membrane opening (BMO), border tissues, and scleral opening; however, it is complicated and requires expensive OCT devices. A more popular and simple method has been used in other studies, which is the tilt ratio using fundus image [15,16,17]. The tilt ratio (or ovality index) is usually defined by the ratio between the two maximum diameters of the ONH: the vertical/horizontal or the horizontal/vertical diameter. This method requires only two diameters to be measured in a fundus image, making it an easy and inexpensive examination.

The tilt ratio (or ovality index) is a simple parameter that reflects the amount of actual ONH tilting. Many previous studies have revealed its clinical relevance [15,16,18]; however, several contradictory results have been reported [19,20]. Lee et al. [19] reported that the LC tilt ratio does not correlate with LC tilting. Another study reported that, as myopia progresses, the LC is shifted parallel to the BMO, rather than becoming tilted [20]. According to their report, the ovality of the ONH may actually be a consequence of the shifting of LC and not of tilting.

Therefore, we hypothesized that the tilt ratio reflects not only the ONH tilting but also other factors, such as the LC shifting. The purpose of this study was to develop a new index to represent the ONH tilting without shifting. The new index should be easy to obtain and correlate well with the clinical characteristics of myopic eyes.

## 2. Materials and Methods

This retrospective study was approved by the Institutional Review Board (IRB) of Pusan National University Hospital, South Korea (number 2310-012-132) and conducted according to the Declaration of Helsinki. The requirement for patient consent was waived by the IRB because of the retrospective nature of the study.

A retrospective review of the detailed results of ophthalmic examinations was performed, including best corrected visual acuity (BCVA); Goldmann applanation tonometry (GAT); slit-lamp examination; funduscopy; biometry using the IOL Master (Carl Zeiss Meditec, Dublin, CA, USA); Humphrey visual field test (Carl Zeiss Meditec); central corneal thickness (CCT) using ultrasonic pachymetry (Pachmate; DGH Technology, Exton, PA, USA); and keratometry using the Auto Kerato-Refractometer (ARK-510A; NIDEK, Hiroshi, Japan).

All medical records were obtained from participants who visited the glaucoma clinic at the Pusan National University Hospital between January 2006 and March 2021. The inclusion criteria were eyes with myopia (spherical equivalent < –0.5 dioptres [21,22]); patients aged 18 years or older; and fundus photography clearly readable to determine the ONH boundary. Patients with optic neuropathies other than glaucoma; macular diseases, such as age-related macular degeneration (AMD); recent ocular surgery; or trauma were excluded. One eye was randomly selected if both the eyes were eligible for inclusion.

### 2.1. Measuring Optic Nerve Head Curvature

**Figure 1:**
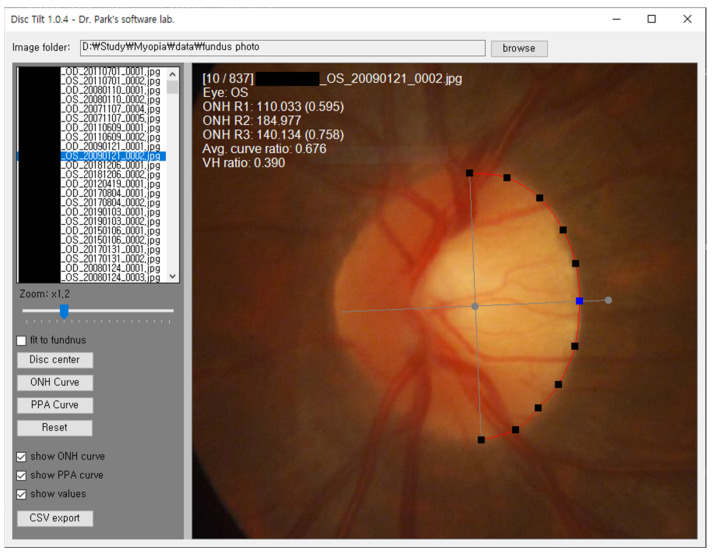
Custom software was developed to measure optic nerve head (ONH) average curvature ratio (ACR) and vertical–horizontal (VH) ratio. Vertical/horizontal axis and 11 points along with ONH margin were determined by two glaucoma specialists. ACR and VH ratio are automatically calculated.

We developed custom Windows software (Figure 1) using Microsoft Visual Studio 2019 and the C# language with a dot net library to measure the ONH curvature. The measurement process began with the user specifying the vertical axis of the ONH. Once the user clicked on both the edges of the vertical axis, the software drew two lines, the vertical and horizontal axis lines, perpendicular to each other. Subsequently, 11 movable points (P1–P11) were initially shown on the vertical axis line, and the user manually dragged them towards the ONH boundary. The average curvature ratio was automatically calculated when the user moved through points P1–P11. These values can be downloaded in the CSV (comma-separated values) file format. Two glaucoma specialists confirmed the ONH boundary points (P1–P11) and vertical/horizontal axis lines.

### 2.2. Calculation of the Average Curvature Ratio, Expected Tilt Angle, and Vertical–Horizontal Ratio

**Figure 2:**
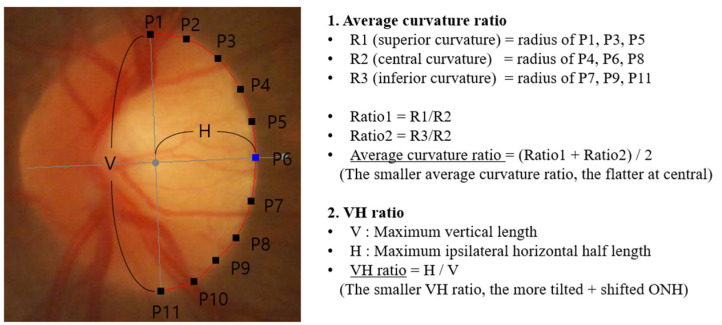
Calculation of average curvature ratio (ACR) and vertical–horizontal (VH) ratio.

Figure 2 shows the calculations of two important parameters in this study. To obtain the average curvature ratio (ACR), the first step was to calculate the superior/central/inferior curvatures, which are defined by the radius of a circle passing through (P1, P3, P5)/(P4, P6, P8)/(P7, P9, P11). Two curvature ratios were then calculated by dividing the superior or inferior curvature by the central curvature. Finally, the average of the two curvature ratios was calculated as the ACR. The lower the ACR, the flatter the ONH at center.

**Figure 3:**
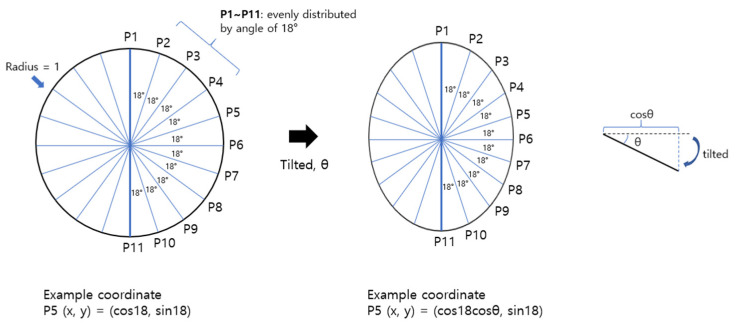
Calculation of coordinates P1~P11 on imaginary circle (radius = 1) for the expected tilt angle.

Figure 3 shows the calculation of the expected tilt angle. The expected tilt angle was defined as the angular ONH tilt presumed by the ACR. If there is a circle of radius 1, and 11 points (P1–P11) are placed on half of the circle evenly distributed at an angle of 18°, the coordinates of each point can be calculated using trigonometric functions. For example, P6 = (1, 0); P5 = (cos18, sin18); and P4 = (cos36, sin36). When this circle is tilted at an angle of θ, the axis X of each point will be shortened by cos θ while the axis Y will not change. Thus, the coordinates will be P6 = (cos θ, 0); P5 = (cos18 cos θ, sin18); P4 = (cos36 cos θ, sin36); and so on. Once all the coordinates of P1~P11 are calculated, we can obtain the ACR at the tilt angle θ. In this way, we can pre-calculate the ACR values at angles of 0°, 1°, 2°, … 89°. The expected tilt angle was determined by comparing the actual ACR with the pre-calculated ACR and selecting the best matched ACR.

Figure 2 shows the calculation of the VH ratio. This is the simple ratio of the vertical and horizontal axes lengths. The vertical axis was defined as the longest line perpendicular to the direction of the ONH tilting. The horizontal axis was defined as the longest line perpendicular to the vertical axis. The VH ratio decreased when the vertical length was greater than the horizontal length. Both shifting and tilting of ONH can make the ONH vertically elongated and the VH ratio becomes smaller. The ONH shifting is defined as horizontal movement (translation) of scleral opening relative to the BMO.

### 2.3. Visual Field Examination and Definition of Glaucomatous Scotoma

**Figure 4:**
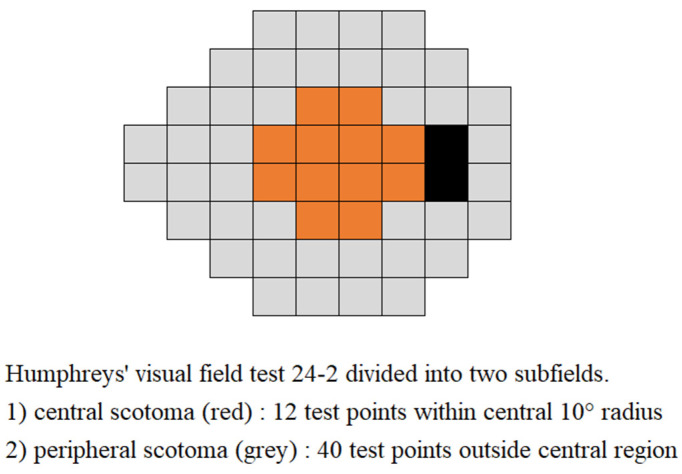
Definition of central and peripheral scotoma.

Within 6 months of the fundus photo examination, automated perimetry was performed on all patients using a Humphrey Visual Field Analyzer 750i instrument (Carl Zeiss Meditec) with the Swedish Interactive Threshold Algorithm (SITA) 24-2 or 30-2. The 30-2 test pattern was converted to 24-2 using overlapping test points. Reliable visual field tests were defined as having a false-positive rate < 30% and a false-negative rate < 30%. The fixation loss was not considered because it has little impact on reliability [23]. Glaucomatous scotoma in the central and peripheral regions (Figure 4) was defined by two criteria:

(1) Patella–Anderson criteria [24]: in each central or peripheral region, a cluster of three or more points had a *p*-value < 5%, one of which had a *p*-value < 1% for the pattern deviation probability map.
(2) Kook’s criteria [25]: in each central or peripheral region, three or more adjacent points with a *p*-value < 5% or two or more test points with a *p*-value < 2% or less on a pattern deviation probability map.

### 2.4. Statistical Analyses

The Shapiro–Wilk test was used to check the normality of the data distribution. To compare the parameters between patient groups, we used the Student’s *t*-test or the Mann–Whitney U test, depending on the normality of the data. The chi-squared test was used for categorical variables. For conducting statistical analyses, R statistics (version 4.0.3 for Windows) was used, and *p* < 0.05 was considered statistically significant.

## 3. Results

A total of 375 eyes of 375 participants were included in this study. The patients were divided into two groups according to the ACR: flat ONH group (ACR < 1.0), and convex ONH group (ACR ≥ 1.0). The demographic characteristics are summarized in Table 1. Of the 375 eyes, 231 (61.6%) were included in the flat ONH group and 144 (38.4%) were included in the convex ONH group. The mean ACR was 0.74/1.30 (flat ONH/convex ONH group). In the flat ONH group, the average expected tilt angle was 32.19 ± 15.38 degrees. Age, sex, spherical equivalent refractive error, intraocular pressure, axial length, and central corneal thickness were not significantly different between the two groups. No visual field global indices (mean deviation, pattern standard deviation, and visual field index) were significantly different between the two groups.

**Figure 5:**
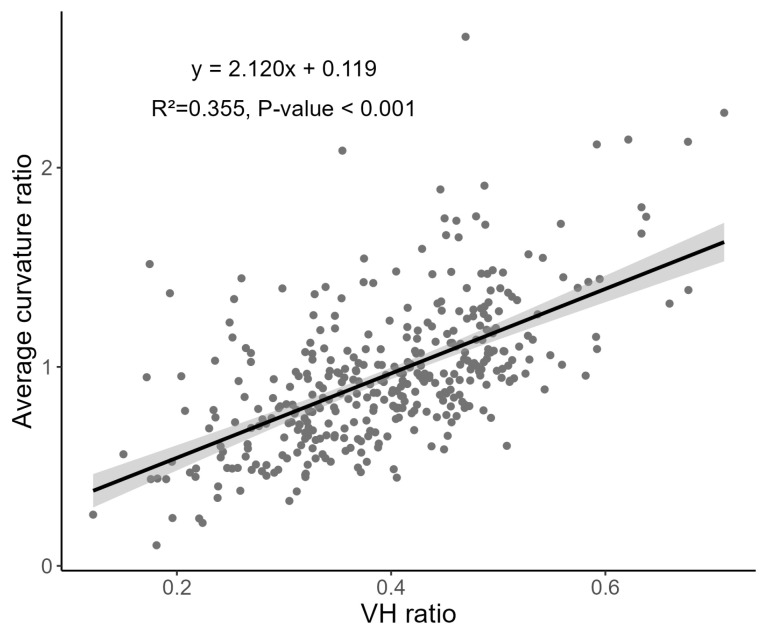
Scatter plots of average curvature ratio (ACR) versus vertical–horizontal (VH) ratio. ACR was significantly correlated with VH ratio, which means that when optic nerve head (ONH) shifting occurs, tilting of the ONH also occurs.

**Figure 6:**
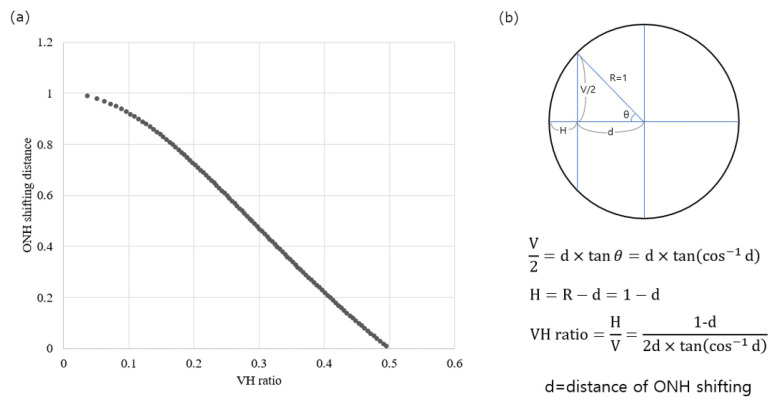
Calculation of vertical–horizontal (VH) ratio according to distance of optic nerve head (ONH) shifting in an imaginary circle (radius = 1). (**a**) The smaller VH ratio, the larger ONH shifted. (**b**) Calculation formula.

A scatter plot of the ACR versus the VH ratio is shown in Figure 5. The ACR was significantly associated with the VH ratio (R^2^ = 0.355, *p* < 0.001). As the VH ratio decreased, the ACR also tended to decrease. Figure 6 shows our calculation of VH ratio according to distance of ONH shifting in imaginary circle. As the VH ratio decreased, ONH was shifted more. This means that the ONH shifting and tilting do not occur independently but are associated with each other.

**Figure 7:**
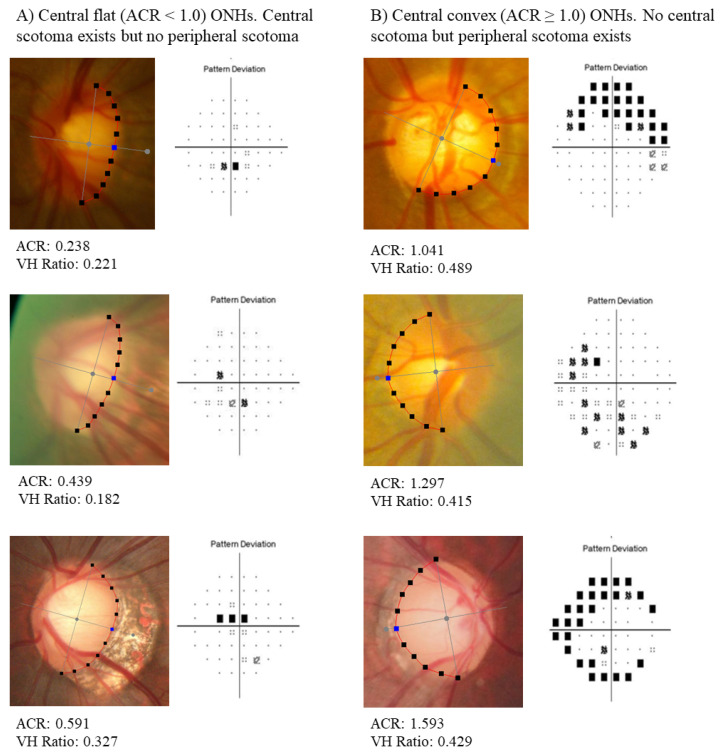
Representative cases of fundus photo and visual field exam.

As shown in Table 2, central scotoma occurred more frequently in the flat ONH group, whereas peripheral scotoma did not differ significantly between the groups (representative cases are shown in Figure 7). The number of eyes with central scotoma, as defined by the Patella–Anderson criteria, was 103 (44.6%) in the flat ONH and 44 (30.6%) in the convex ONH group, which was significantly different (*p* = 0.009). A similar tendency was observed when Kook’s criteria were applied. The number of eyes with central scotoma was 122 (52.8%) in the flat ONH and 50 (34.7%) in the convex ONH group, which was also significantly different (*p* < 0.001). However, peripheral scotoma did not differ between the flat and convex ONH groups. The number of eyes with peripheral scotoma according to the Patella–Anderson criteria was 140 (60.6%) in flat ONH and 87 (60.4%) in the convex ONH group (*p* = 1.000), and according to Kook’s criteria, 161 (69.7%) in the flat ONH and 98 (68.1%) in the convex ONH group (*p* = 0.826).

**Figure 8:**
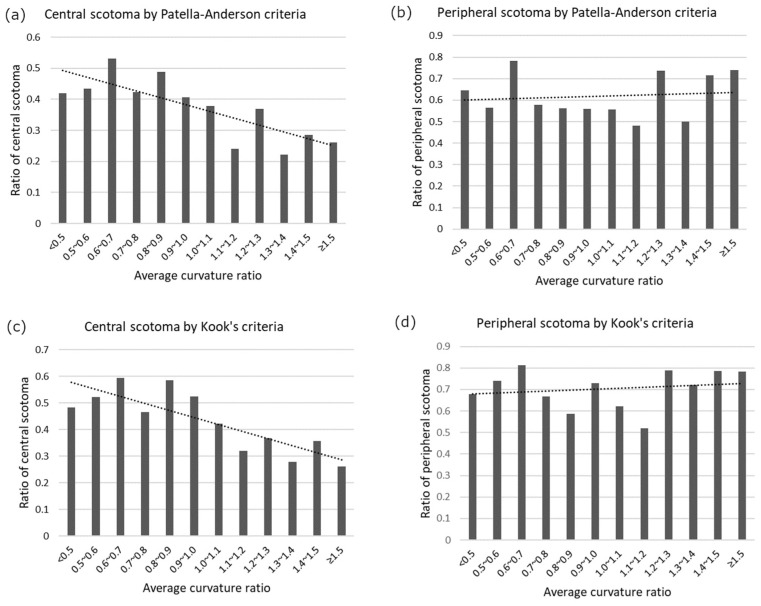
Central/peripheral scotoma ratio binned by average curvature ratio (ACR). To define the glaucomatous scotoma, Patella–Anderson criteria (**a**,**b**) and Kook’s criteria (**c**,**d**) were used. The central scotoma ratio became larger as the ACR decreased, which means the more centrally flattened optic nerve head, the more central scotomas occur. In contrast, peripheral scotoma ratio showed little changes as ACR increased.

A similar tendency is observed in Figure 8. Bar plots show the ratio of central/peripheral scotomas bound by ACR. The ratio of central scotoma decreased as the ACR increased (Figure 8a,c). Specifically a centrally flatter ONH (smaller ACR) was associated with more frequent central scotoma. However, the ratio of peripheral scotomas changed slightly as the ACR increased. This indicates that peripheral scotoma occurs regardless of the ACR.

Table 3 shows the Spearman’s correlation analysis between the ACR and various factors. ACR was positively correlated with spherical equivalence (ρ = 0.158, *p* = 0.005), which means that as myopia becomes worse, the ONH becomes more tilted and flatter. The axial length was not significantly correlated with ACR (ρ = –0.098, *p* = 0.075); however, its *P*-value was almost close to 0.05. The central corneal thickness was not significantly correlated with ACR (ρ = 0.050, *p* = 0.476)

## 4. Discussion

The main objective of this study was to develop a new index, ACR, as a surrogate measurement of ONH tilting and determine its clinical relevance. In the flat ONH group, the mean ACR was 0.74 ± 0.19 and the expected tilt angle was 32.19°. Patients with a lower ACR (more tilted ONH) had central scotoma more frequently; however, peripheral scotoma was not associated with ACR. The ACR was positively correlated with spherical equivalence, which means that the higher the myopia, the more tilted the ONH. The ACR was not correlated with the axial length or central corneal thickness.

Previous studies have demonstrated that myopic eyes undergo various changes in shape. Greene [26] reported that extraocular muscles, especially oblique muscles, exert mechanical stresses on the posterior sclera in both the axial and tangential directions, causing the myopic eye to change its shape. In their report, the effects of tensile stress varied significantly according to the muscular attachment site, muscle strength, and scleral thickness. Another study reported that the adduction of the eye may also induce ONH tilting [27]. In eyes with a shorter length of optic nerve, a large adduction may exhaust the length redundancy of optic nerve and stretch the optic nerve and deform the eyeball. Myopic elongation is associated not only with the changes in shape but also with various tissue changes [8]. It includes a decrease in photoreceptor cells, choroidal and scleral thinning, and a shift in BMO.

This mechanical stress induces ONH tilting and torsion in myopic eyes and makes them susceptible to the development of glaucoma [3,28,29]. In previous studies, the “tilt ratio”, which is similar to our VH ratio, was frequently used to measure the extent of ONH tilting. The tilt ratio is usually defined as the ratio of the disc diameter, longest/shortest or shortest/longest diameter. Sawada et al. [15] reported that eyes with a worse VF had a significantly greater tilt ratio. Hong et al. [5] also reported that a greater tilt ratio was significantly correlated with a worse central VF. Choi et al. [16] reported that the horizontal ONH tilt (temporally tilted ONH) and the angular location of the maximal ONH tilt were associated with the severity and location of VF defects in myopic glaucoma. What these studies have in common is that a more tilted ONH is associated with worse VF damage, and the direction of tilting is associated with the location of VF damage. As the main direction of ONH tilting is known to be the temporal side [17], central VF damage is probably more frequent when the ONH is tilted. This is consistent with our study. Instead of using the tilt ratio, we used our new index, ACR, to represent the ONH tilting, which was significantly associated with central VF damage but not with peripheral VF damage.

In addition to tilting and torsion, another type of ONH distortion has been observed. In a longitudinal study, Lee et al. [20] reported that the LC shifted nasally during myopia progression. An interesting finding in their study was that the LC was mostly shifted rather than tilted. The oval appearance of the ONH was a result of LC shifting, but not tilting. If this theory is correct, the tilt ratio (or ovality index) does not represent the ONH tilting but the amount of shifting. However, we believe that this was not the case. If only shifting occurs, the change in ONH curvature cannot be explained. We found that ACR was significantly correlated with the VH ratio (Figure 2). If the ONH is not tilted but is only shifted, the VH ratio is only related to shifting, and the ACR should remain unchanged, as the VH ratio changes because the ONH curvature remains unchanged during shifting. We presume that both shifting and tilting occur simultaneously, and that the tilt ratio reflects both. Thus, the tilt ratio partially reflects the amount of ONH tilting, and sometimes it may not be correct, especially when shifting is more dominant than tilting.

There is evidence that the tilt ratio (or ovality index) does not correlate with actual ONH tilting. Lee et al. [19] investigated the relationship between the tilt ratio and the LC tilt angle using OCT. In their study, the tilt ratio was not associated with the LC tilt angle. Moreover, some ONHs with large tilt ratios exhibit minimal LC tilt angles without VF defects. Although the tilt ratio did not correlate with the LC tilt angle, a large LC tilt angle measured using OCT was significantly related to the presence of glaucoma. They concluded that the angulation of the LC might be associated with an increased susceptibility to glaucoma. The results of this study support these hypotheses. The tilt ratio partially reflects the actual ONH tilt and is sometimes incorrect. The tilt ratio is affected by ONH tilting and shifting.

**Figure 9:**
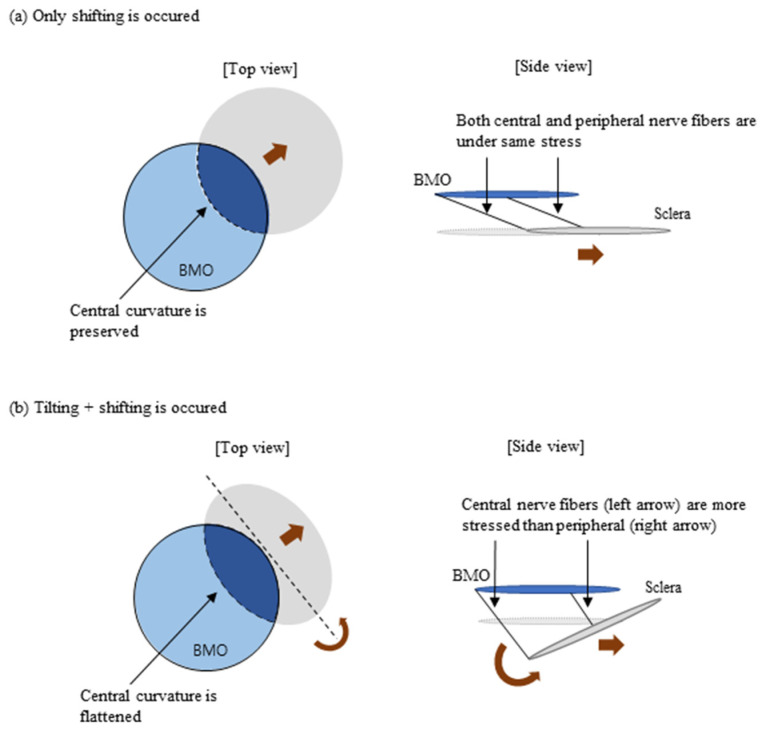
Shifting versus tilting. (**a**) Illustrates the case when only a horizontal shifting of the optic nerve head (ONH) occurs. The relative position of the scleral opening (grey circle) to BMO (blue circle) is shifted. In this case, the central curvature of ONH does not change while the VH ratio becomes smaller. Both central and peripheral nerve fibers will be evenly stressed because the elongated length are the same. (**b**) Illustrates the case when both tilting and shifting of the ONH occurs. Scleral opening is not only horizontally shifted but also rotated (tilted). In this case, the central curvature becomes flattened because of rotation, and this makes the average curvature ratio (ACR) smaller. The VH ratio, which is affected by both tilting and shifting, also becomes smaller. BMO: Bruch’s membrane opening.

A tilted ONH is important because it is associated with papillomacular bundle damage. Kim et al. [18] reported that in children with myopic shift, the ONH progressively tilted and shifted in the nasal direction. Kimura et al. [28] also reported that the tilting of the ONH may induce a tensile stretching of the temporal side of the LC and papillomacular bundle. In this regard, ONH tilting is important because it is associated with papillomacular bundle damage and subsequent central VF scotoma. However, the problem with the tilt ratio is that it is coupled with both ONH tilting and parallel shifting. Parallel shifting of the ONH not only places stress on the papillomacular bundle, but also exerts equal stress on the peripheral nerve fibers (Figure 9). However, we were more interested in the amount of ONH tilting because it places a more centrally directed stress on the ONH (Figure 9). We propose that the ACR is a good surrogate for representing the amount of ONH tilting; unlike the tilt ratio, the ACR may be affected by ONH tilting alone.

Studies have shown that the ONH tilting is negatively correlated with the refractive error. Hyung et al. [30] reported that a higher refractive error correlated with a larger tilt ratio. Lim et al. [31] reported that a tilted ONH had significantly more myopic refractive error than eyes without a tilted ONH. However, the relationship between axial length and ONH tilting remains controversial. Lim et al. [31] and Han et al. [32] reported a positive correlation between longer axial length and larger ONH tilting. However, other studies have not found a significant correlation [33,34]. These results are consistent with our findings. The ACR in our study positively correlated with spherical equivalence, which means that the more tilted the ONH, the more myopic the refractive error. The ACR was not significantly correlated with the axial length (*p* = 0.053); however, the *p*-value was very close to the significance level.

The limitation of this study is that we did not investigate the correlation between actual tilting measured by OCT and ACR. We are planning a further study using swept-source OCT to measure actual ONH tilting and to prove that ACR reflects it.

## 5. Conclusions

The amount and direction of ONH tilt are important in myopic eyes because they are associated with central VF defects. The tilt ratio (or ovality index) has been frequently used in previous studies to represent the amount of tilting. However, the tilt ratio is affected by ONH tilting and shifting. Therefore, it is a partial and incomplete index. The new ACR index in our study reflects the amount of ONH tilting and is a good index for estimating the central VF damage.

## Figures and Tables

**Table 1:** Demographic characteristics.

	Flat ONH ^1^	Convex ONH ^2^	*p*-Value
Number of eyes	231 (61.6%)	144 (38.4%)	
Average curvature ratio	0.74 ± 0.19	1.30 ± 0.29	<0.001 ^3^
Age (years)	39.8 ± 12.2	41.4 ± 12.5	0.276 ^4^
Gender (male/female)	121/110	74/74	0.546 ^5^
Refractive error (spherical equivalent, Diopter)	–5.64 ± 2.69	–5.22 ± 2.42	0.173 ^3^
Intraocular pressure (mmHg)	17.0 ± 4.5	16.4 ± 5.3	0.212 ^3^
Axial length (mm)	26.07 ± 1.48	25.93 ± 1.55	0.546 ^4^
Central corneal thickness (μm)	555.2 ± 35.8	557.3 ± 50.1	0.234 ^4^
Visual field global indices			
-Mean deviation (dB)	–5.89 ± 6.06	–5.72 ± 6.39	0.793 ^3^
-Pattern standard deviation (dB)	5.60 ± 4.65	4.85 ± 4.23	0.119 ^3^
-Visual Field Index (%)	86.8 ± 18.04	87.52 ± 19.25	0.713 ^3^

Values are presented as mean ± standard deviation. ONH: optic nerve head. ^1^ Patients with average curvature ratio < 1.0; ^2^ Patients with average curvature ratio ≥ 1.0; ^3^ Student’s *t*-test; ^4^ Mann–Whitney U test; ^5^ χ^2^ test.

**Table 2:** Number of eyes with central/peripheral scotoma according to the optic nerve head (ONH) shape.

	Flat ONH (N = 231)	Convex ONH (N = 144)	*p*-Value ^1^
Central scotoma (no. of eyes)			
By Patella–Anderson criteria	103 (44.6%)	44 (30.6%)	0.009
By Kook’s criteria	122 (52.8%)	50 (34.7%)	<0.001
Peripheral scotoma (no. of eyes)			
By Patella–Anderson criteria	140 (60.6%)	87 (60.4%)	1.000
By Kook’s criteria	161 (69.7%)	98 (68.1%)	0.826

^1^ χ^2^ test.

**Table 3:** Correlation analysis between the average curvature ratio (ACR) and various factors.

Factors	Rho ^1^	*p*-Value ^1^
Spherical equivalence	0.158	0.005
Axial length	–0.098	0.075
Central corneal thickness	0.050	0.476

^1^ Spearman’s correlation analysis.

## Data Availability

The data that support the findings of this study are available from the corresponding author.
